# Interactions of Oligodendrocyte Precursor Cells and Dopaminergic Neurons in the Mouse Substantia Nigra

**DOI:** 10.1111/jnc.16298

**Published:** 2025-01-27

**Authors:** Julia C. Fitzgerald, Ying Sun, Frederek Reinecke, Elisabeth Bauer, Olga Garaschuk, Philipp J. Kahle, Friederike Pfeiffer

**Affiliations:** ^1^ Hertie Institute for Clinical Brain Research University of Tübingen Tübingen Germany; ^2^ Institute for Physiology University of Tübingen Tübingen Germany; ^3^ The German Centre for Neurodegenerative Diseases Tübingen Germany; ^4^ Institute of Biochemistry University of Tübingen Tübingen Germany

**Keywords:** dopaminergic neurons, midbrain, mouse brain, oligodendrocyte precursor cells, OPCs, Parkinson's disease, SNpc

## Abstract

Parkinson's disease (PD) is a prevalent neurodegenerative disease caused by the death of dopaminergic neurons within the *substantia nigra pars compacta* (SNpc) region of the midbrain. Recent genomic and single cell sequencing data identified oligodendrocytes and oligodendrocyte precursor cells (OPCs) to confer genetic risk in PD, but their biological role is unknown. Although SNpc dopaminergic neurons are scarcely or thinly myelinated, there is a gap in the knowledge concerning the physiological interactions between dopaminergic neurons and oligodendroglia. We sought to investigate the distribution of OPCs with regard to the myelination state in the mouse *substantia nigra* (SN) by high‐resolution imaging to provide a morphological assessment of OPC‐dopaminergic neuron interactions and quantification of cell numbers across different age groups. OPCs are evenly distributed in the midbrain throughout the lifespan and they physically interact with both the soma and axons of dopaminergic neurons. The presence of OPCs and their interaction with dopaminergic neurons does not correlate with the distribution of myelin. Myelination is sparse in the SNpc, including dopaminergic fibers originating from the SNpc and projecting through the *substantia nigra pars reticulata* (SNpr). We report that OPCs and dopaminergic neurons exist in a 1:1 ratio in the SNpc, with OPCs accounting for 15%–16% of all cells in the region across all age groups. This description of OPC‐dopaminergic neuron interaction in the midbrain provides a first look at their longitudinal distribution in mice, suggesting additional functions of OPCs beyond their differentiation into myelinating oligodendrocytes.

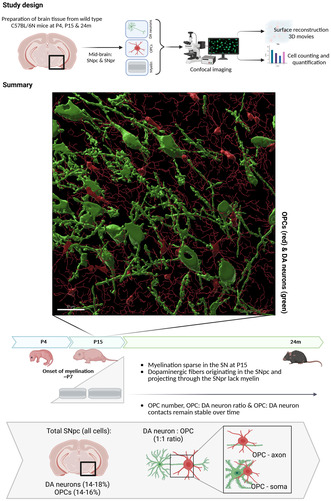

## Graphical abstract

In this study, we analyzed the ratio and interaction between oligodendrocyte precursor cells (OPCs) and dopaminergic neurons (DA neurons) in the substantia nigra pars compacta (SNpc) and reticulata (SNpr), a brain region that is sparse in myelin. We found that both DA neurons and OPCs comprise a constant portion of cells (14%–18%) in the SN across all age groups analyzed, newborn, juvenile, and aged mice. Notably, OPCs closely interacted with the soma and processes of DA neurons, and these interactions also remained stable over time, indicating an important role for OPCs in the SN independent of the occurrence of myelin.

**Abbreviations**
6‐OHDA: 6‐hydroxydopamine
CNS: central nervous system
cp: cerebellar peduncle
DA: dopamine
DAPI: 4′,6‐diamidino‐2‐phenylindole
GABA: γ‐aminobutyric acid
LC: locus ceruleus
MBP: myelin basic protein
ml: medial lemniscus
MRI: magnetic resonance imaging
NeuN: neuronal nuclear protein
NG2: nerve/glial antigen 2
OPC: oligodendrocyte precursor cell
PBP: parabrachial pigmented nucleus
PBS: phosphate‐buffered saline
PD: Parkinson's Disease
PDGFRα: platelet‐derived growth factor alpha
PFA: paraform aldehyde
RNA: ribonucleic acid
scp: superior cerebellar peduncle
SN: substantia nigra
SNpc: substantia nigra pars compacta
SNpr: substantia nigra pars reticulata
TH: tyrosine hydroxylase
VTA: ventral tegmental area

## 1 Introduction

Parkinson's disease (PD) is the fastest‐growing neurodegenerative disorder worldwide yet there are currently no drugs available that can halt or prevent the onset of this movement disorder. One reason for the lack of effective disease‐modifying therapies is that by the time patients experience first motor symptoms, a substantial proportion of dopaminergic neurons in the SNpc have already degenerated.

Several studies combining large datasets for genetic risk and transcriptional profiling have highlighted multiple cell type involvement in the selective degeneration of the dopaminergic neurons in PD. Data from single‐cell sequencing in the SN of healthy *postmortem* brain tissue identified a distinct cell type association between genetic risk to develop PD and oligodendrocyte‐specific gene expression (Agarwal et al. 2020). Another study concluded that PD risk loci lie across several cell types including astrocytes and oligodendrocytes (Reynolds et al. 2019). Using genome‐wide association study data combined with Braak disease staging (Braak et al. 2003), Bryois et al. (2020) suggested that genetic risk for PD converges to the oligodendroglia and the ensuing changes could proceed dopaminergic degeneration. Feleke et al. (2021) combined single nuclei and bulk tissue RNA sequencing to profile molecular pathologies in Lewy body diseases including PD and found that differentially expressed genes in glia enrich for heritability of PD age of onset and genetic risk. A recent paper by Martirosyan et al. (2024) provides a single nuclei RNA‐seq dataset of human post‐mortem SNpc samples and describes the constitution of the human SNpc as ~41% oligodendrocytes, followed by astrocytes (~25%), microglia (~15%), neurons (~7%), OPC (~8%), and T cells (< 2%). Another study estimated the OPC population of the human SNpc to be ~6.5% (Wang et al. 2024). In the PD SNpc, Martirosyan et al. (2024) found an increase in the relative proportion of glial and T cells and a decrease in the number of neurons. Wang et al. (2024) also report an increase in OPCs in the SNpc compared to the control brain. There is a scientific need to further characterize the role of oligodendroglia and their subpopulations in the midbrain, specifically their morphology and interaction with dopaminergic neurons.

Oligodendrocytes are the myelinating cells of the central nervous system (CNS). The myelin sheath supports the underlying axon and influences its conductive properties (Saab and Nave 2017). It has become apparent that oligodendrocytes are affected in several neurodegenerative diseases (Festa et al. 2024). In PD patients, MRI scans have shown overall alterations in the white matter, with the majority of connections with reduced myelin content emerging from the basal ganglia (Boshkovski et al. 2022). Oligodendrocytes are generated from oligodendrocyte precursor cells (OPCs). In rodents, OPC proliferation peaks in the first two postnatal weeks. During this period, their number greatly increases, and they migrate and distribute throughout the entire CNS, in white as well as gray matter (Nishiyama et al. 2021). Paradoxically, dopaminergic neurons localized in the SNpc have notably long axons, yet for reasons unknown, are scarcely myelinated (Braak and Del Tredici 2004; Orimo et al. 2011; Sulzer and Surmeier 2013). This may in part contribute to the vulnerability of dopaminergic neurons but does not explain the specific degeneration of SNpc dopaminergic neurons in PD. Interestingly, a recent study showed myelination of the VTA dopaminergic neurons can be modulated in response to neuronal activity evoked by optogenetic stimulation (Yalcin et al. 2024).

Despite the recently appreciated contribution of the oligodendroglia in PD, many questions regarding their functions in the *substantia nigra* (SN) in health and the development of PD remain. In this context, we focus on OPCs, the non‐myelinating oligodendrocyte precursors.

The distribution of OPCs in the midbrain and whether they interact with dopaminergic neurons is not well morphologically described. We therefore assessed the spatial distribution of OPCs in the mouse SN at different ages, including a very early developmental time point before the onset of myelination and counted OPCs and dopaminergic neurons in the SNpc of mice at different ages.

## 2 Methods

### 2.1 Mouse Brain Tissue Preparation

C57BL/6 mice were bred in type II long cages. After weaning at day 21, male and female mice were kept separately in groups of up to 5 mice per cage. Mice had access to food and water at any time.

At P4, C57BL/6 mice were decapitated and the brains were removed. At P15, C57BL/6 mice were anesthetized with an intraperitoneal injection of Ketamine 200 mg/kg body weight/Xylazine 20 mg/kg body weight and transcardially perfused with 4% PFA (EM‐grade, Science Services) containing 0.1 M L‐lysine and 0.01 M sodium metaperiodate (described by (Pfeiffer, Sherafat, and Nishiyama 2021)) or 4% Formaldehyde (ROTI Histofix, Carl Roth, P087.1). Brains were dissected and post‐fixed for 1–2 h or overnight in the same fixative and then washed in 0.2 M sodium phosphate buffer/phosphate‐buffered saline (PBS), pH 7.4, and cryoprotected in 30% sucrose/0.2 M sodium phosphate buffer or PBS before being snap‐frozen in Tissue Tek (OCT compound, Sakura) on Isopentane/dry ice and stored at −80°C. The C57BL/6 24‐month‐old mice recommended for euthanasia due to illness/strong aging were anesthetized with 5%–10% isofluorane (until upright reflex subsided completely), cervically dislocated, and decapitated. The brain is extracted from the skull and washed in PBS. Mouse brains were fixed in 4% PFA for 2–3 h and immersed in 30% sucrose/PBS for at least 24 h before being embedded in Tissue Tek and frozen as described above. All experiments were performed in accordance with the German Animal Welfare Law and the allowance of the local authorities (Regierungspräsidium Tübingen).

### 2.2 Immunohistochemistry

50 μm thick sections from brains taken at P4 and P15 were cut with a Leica CM1950 cryotome. (For the brains from 24‐month‐old mice: 20 μm cryosections were cut with a Leica CM3050S cryotome, directly mounted on SuperFrost microscopy slides (Langenbrinck), stored at −20°C until usage, air‐dried, and slices circles with PapPen). Free‐floating sections or slices mounted on glass slides were rinsed in PBS and blocked with 1% bovine serum albumin (BSA) and 1% Triton X‐100 in phosphate‐buffered saline (PBS, Sigma Aldrich) for 1 h at room temperature before being incubated with primary antibodies (goat anti‐PDGFRα, R&D Systems AF1062, diluted 1:250, mouse anti‐Tyrosine‐Hydroxylase, Millipore MAB318, diluted 1:400, rat anti‐Myelin Basic Protein (MBP), Abcam ab7349, diluted 1:250, rabbit anti‐Sox10, Abcam ab227680, diluted 1:200, rabbit anti‐neuN, Millipore ABN78, diluted 1:500). The next day, sections were rinsed 3x in PBS followed by incubation with secondary antibodies (donkey anti‐mouse Alexa Fluor 488, Invitrogen A21202; donkey anti‐goat Alexa Fluor 488, Invitrogen A11055; donkey anti‐goat Alexa Fluor 594, Invitrogen A11058; donkey anti‐rat Alexa Fluor 594, Invitrogen A21209; donkey anti‐mouse Alexa Fluor 647, Jackson Immuno Research 715‐605‐151; donkey anti‐rabbit Alexa Fluor 647, Jackson Immuno Research 711‐605‐152; all diluted 1:1000) for 1–2 h at room temperature Table 1. Sections were rinsed again and mounted with Vectashield mounting medium (Vector Laboratories). Nuclei were stained with DAPI (4′,6‐Diamidin‐2‐phenylindol, Dihydrochloride, Thermo Fisher Scientific) or Sytox Deep Red Nucleic Acid Stain (Thermo Fisher Scientific).

### 2.3 Fluorescence Microscopy

Images were acquired using a Leica SP8 confocal microscope (Leica Microsystems, Germany) or Zeiss Apotome (Carl Zeiss AG, Germany). Tile scans were acquired using a 40x objective and areas were defined using the LasX software (Leica Microsystems). High‐resolution images were acquired with 40× or 63× objectives. Z‐step size was 0.35 μm.

### 2.4 Image Projections and 3D Reconstruction

z‐projections were generated with Image J2 (Fiji) (average intensity). 3D reconstructions were generated with Imaris 10.0 (Oxford Instruments, UK). After converting the lif‐files, a surface was generated for the green (TH) and red (OPC) channels using the surface function. Movies (mp4) were generated using the animation tool of Imaris. For the movies, neurons were reconstructed with the surface tool, and OPCs were reconstructed with the filament tool. The graphical abstract was generated in BioRender.

### 2.5 Cell Counting and Statistics

For P4, the brains of 3 mice were analyzed. For P 15, the brains of 6 mice were analyzed. For P24 months, the brains of 4 mice were analyzed. The sample size for the statistical assessment of cell numbers was calculated with G*Power v3.1.9.7 (Faul et al. 2007). In order to detect a decrease in the percentage of TH+ neurons of 20% (which would be a significant change, but below the loss of neurons necessary to cause deficits in movement) with a standard deviation of 5%, a significance of 5% and the power of 80%, 3 mice per group are required for the statistical analysis to detect differences between independent means. After converting the lif files, single‐plane image processing and automated cell counting were performed on confocal‐acquired 3D stacks using Imaris 9.80 (Oxford Instruments, UK) with the spot detection algorithm tool. In each age group, three mice were included. For each mouse, five images from the midbrain SNpc were acquired for cell counting. Images from the P4 and P15 age groups were standardized to a size of 1024 × 1024 pixels, meanwhile, images from the P24 months age group were 1388 × 1040 pixels. Following automatic counting by IMARIS 9.80, all images were manually inspected to obtain the final cell numbers. Statistical analysis was then performed with GraphPad Prism 10 (GraphPad Software, USA). Normality was tested by applying the Kolmogorov–Smirnov test, differences among means were assessed with ANOVA. No test for outliers was performed.

**TABLE 1:** Research resource identifiers.

	Source	Cat. No.	RRID
Mice			
C57BL76N mice	Charles River, own breeding		RRID:IMSR_CRL:027
C57BL76J mice	Jackson Laboratories, own breeding		RRID:IMSR_JAX:000664
Chemicals			
L‐lysine	Sigma Aldrich	L5626	
Paraform‐aldehyde (EM‐grade)	Science Services	E15710	
Paraformaldehyde	Sigma Aldrich	158 127‐500G	
Sodium metaperiodate	Sigma Aldrich	S1878	
DAPI	Thermo Scientific	62 248	
Sytox Deep Red Nucleic Acid Stain	Invitrogen	S11380	
Primary antibodies			
Goat anti‐PDGFRα	R&D Systems	AF1062	RRID:AB_2236897
Mouse anti‐Tyrosine Hydroxylase	Millipore	MAB318	RRID:AB_2201528
Rat anti‐MBP	Abcam	ab7349	RRID:AB_305869
Rabbit anti‐Sox10	Abcam	ab227680	RRID:AB_2927464
Rabbit anti‐NeuN	Millipore	ABN78	RRID:AB_10807945
Secondary antibodies			
Donkey anti‐mouse Alexa Fluor 488	Invitrogen/Molecular Probes	A21202	RRID:AB_141607
Donkey anti‐goat Alexa Fluor 488	Invitrogen/Molecular Probes	A11055	RRID:AB_2534102
Donkey anti‐goat Alexa Fluor 594	Invitrogen/Molecular Probes	A11058	RRID:AB_142540
Donkey anti‐rat Alexa Fluor 594	Invitrogen/Molecular Probes	A21209	RRID:AB_2535795
Donkey anti‐mouse Alexa Fluor 647	Jackson Immuno Research	715–605‐151	RRID:AB_2340863
Donkey anti‐rabbit Alexa Fluor 647	Jackson Immuno Research	711–605‐152	RRID:AB_2492288

## 3 Results

### 3.1 Oligodendrocyte Precursor Cells are in Close Contact With Dopaminergic Neurons in the SN During All Stages of Development

We first assessed the distribution and morphology of OPCs in wild‐type mouse SN at post‐natal Day 4 (P4). We chose the time points for analysis for the following reasons: P4 is before the onset of myelination. At this time point, OPCs proliferation is at its peak, and oligodendrocytes are just appearing (see Figure S1), while myelin sheaths have not been generated yet. We were interested if, at that time, OPCs would interact with the soma of dopaminergic neurons in addition to interact with the axons (in order to prepare myelination) at this time point.

We used platelet‐derived growth factor receptor alpha (PDGFRα) as a marker of OPCs in the brain (Marques et al. 2016; Pfeiffer, Sherafat, and Nishiyama 2021; Sherafat, Pfeiffer, and Nishiyama 2021). Previous work has shown that the OPC population is transcriptionally homogenous (Marques et al. 2016; Tasic et al. 2016; Zeisel et al. 2015) and morphological studies show that the OPC marker PDGFRα extends throughout the OPC processes (Pfeiffer, Sherafat, and Nishiyama 2021). Dopaminergic neurons were stained with an antibody against tyrosine hydroxylase (TH), the gold standard marker for dopaminergic neurons.

**FIGURE 1:**
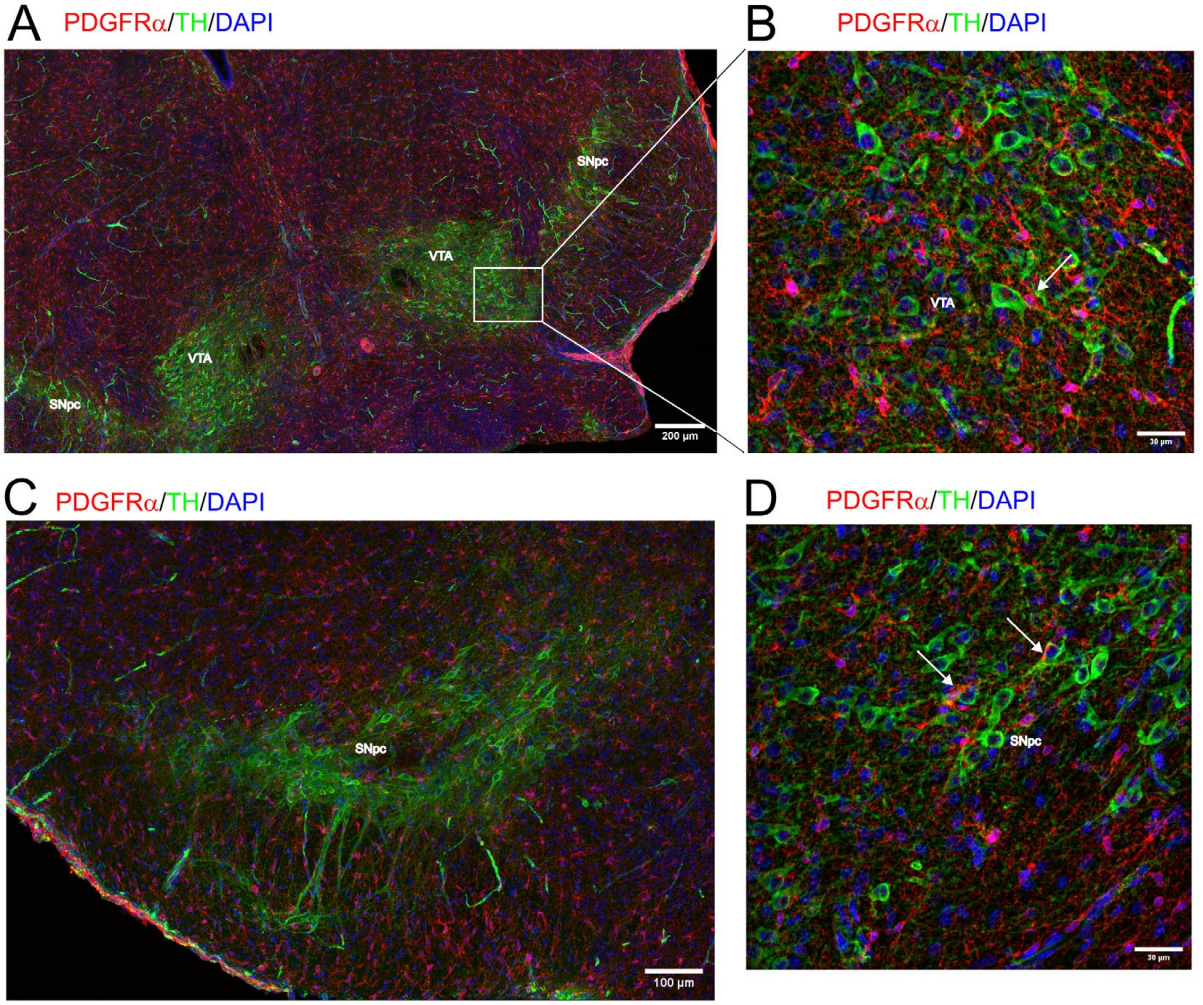
Distribution of OPCs in the midbrain at P4. The distribution of PDGFRα‐positive OPCs (red) is shown in coronal sections of the P4 mouse midbrain. TH+ dopaminergic neurons are shown in green, DAPI+ nuclei are shown in blue. (A) Overview of the midbrain area. Scale bar: 200 μm. (B) Magnification of an area in the VTA shown in A. White arrows indicate close contact between a TH+ dopaminergic‐neuron and an OPC. Scale bar: 30 μm. (C) Overview of the developing substantia nigra in the midbrain. Scale bar: 100 μm. (D) Higher magnification shows OPC processes surrounding the somata of TH+ dopaminergic neurons in the substantia nigra (white arrows). Scale bar: 30 μm.

OPCs were abundantly present throughout the midbrain area (Figure 1A). Tyrosine hydroxylase‐positive dopaminergic neurons accumulate in the *ventral tegmental area* (VTA), (Figure 1B) and the *substantia nigra pars compacta* (SNpc) (Figure 1C,D). The dopaminergic fibers traverse the *substantia nigra pars reticulata* (SNpr) which is known to contain fibers of the dopaminergic neurons (Fu et al. 2012). At higher magnification of the SNpc, close interactions between OPCs and both somata (white arrows) and processes of dopaminergic neurons are observed (Figure 1D). High‐resolution 3D reconstructions of these interactions show that most of the dopaminergic neurons are surrounded by several OPC processes (Movie S1).

**FIGURE 2:**
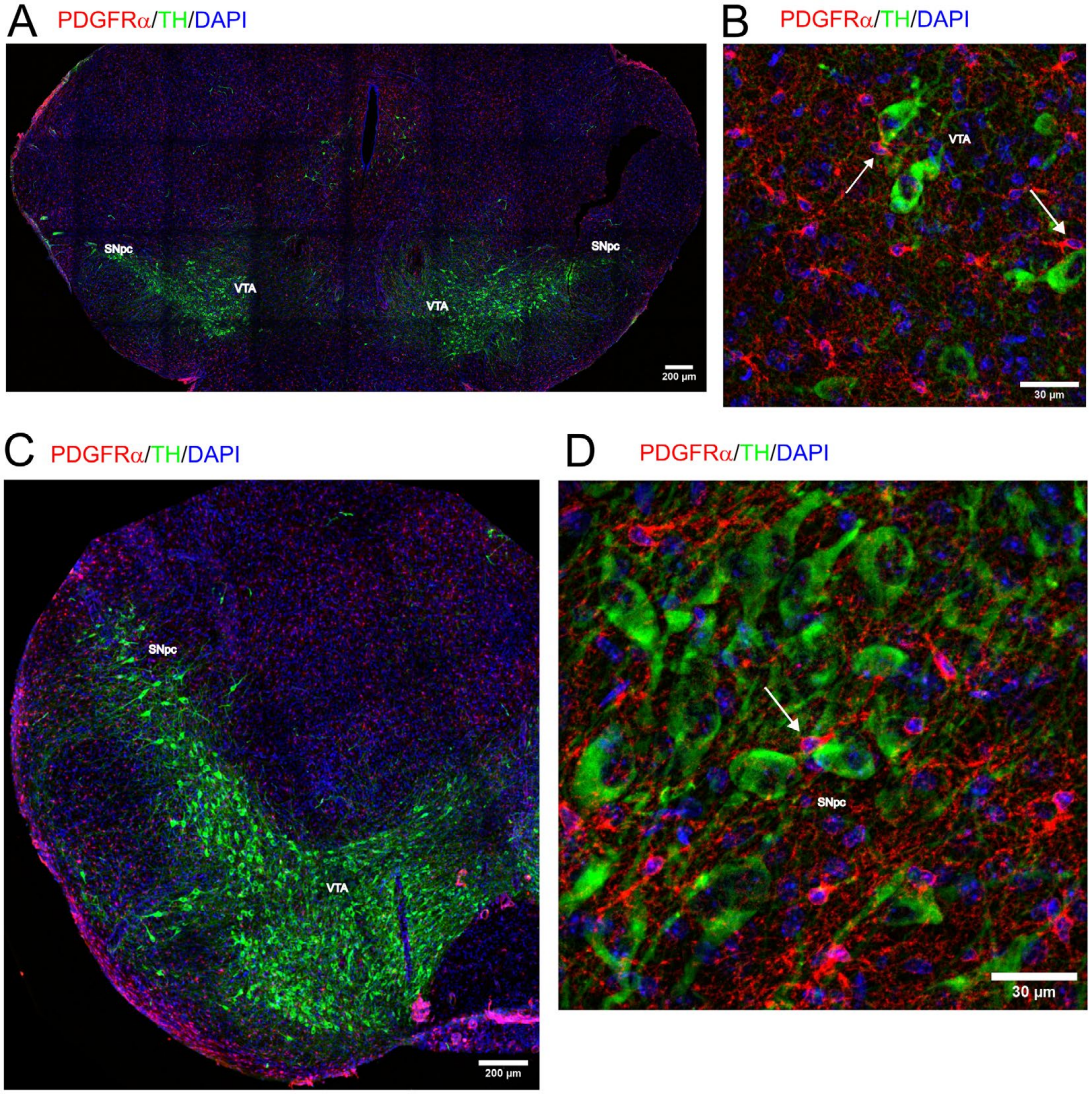
Distribution of OPCs in the midbrain at P15. The distribution of PDGFRα‐positive OPCs (red) is shown in coronal sections of the P15 old mouse midbrain. TH+ dopaminergic neurons are shown in green, DAPI+ nuclei are shown in blue. (A) Overview of the midbrain area. Scale bar: 200 μm. (B) Higher magnification shows OPCs in direct contact with the soma of TH+ dopaminergic neurons (white arrows). Scale bar: 30 μm. (C) Overview of the substantia nigra (SN) and the VTA. Scale bar: 200 μm. (D) Higher magnification shows OPCs intertwined with TH+ dopaminergic neurons (white arrow). Scale bar: 30 μm.

A similar distribution of the dopaminergic neurons and OPCs is seen at P15. At P15, OPC proliferation and density are declining, while oligodendrocyte differentiation as well as myelination reached its peak (Nishiyama et al. 2021). We found that while OPCs are evenly distributed throughout the midbrain (Figure 2A), the dopaminergic neurons accumulate in the SNpc and the VTA (Figure 2C). At higher magnification, close interactions between OPCs and the somata of dopaminergic neurons (white arrows) and their processes are revealed in the VTA (Figure 2B) and the SNpc (Figure 2D). Some OPCs establish contacts with several dopaminergic neurons (Movie S2).

**FIGURE 3:**
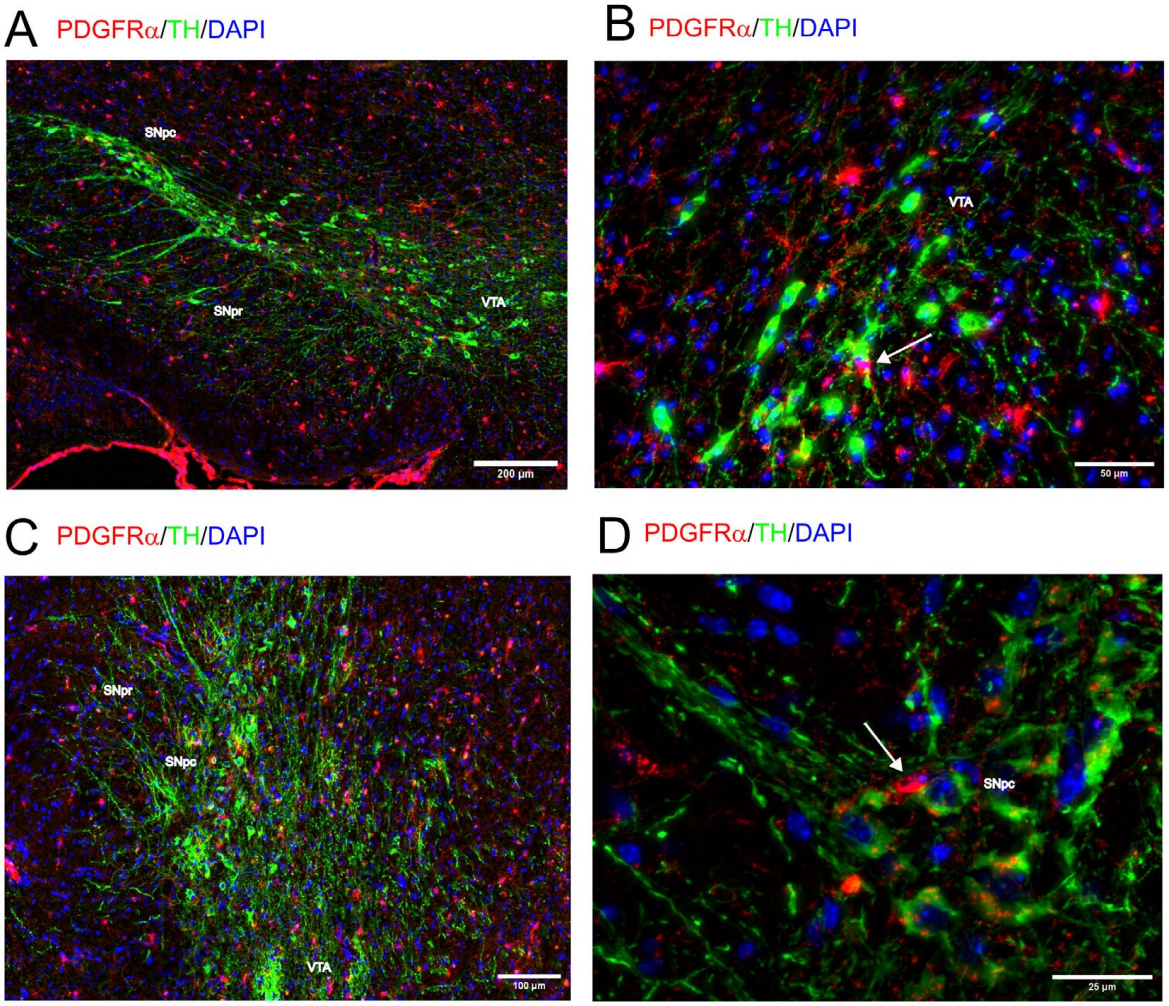
Distribution of OPCs in the midbrain at 24 months. The distribution of PDGFRα‐positive OPCs (red) is shown in coronal sections in the midbrain of 24‐month‐old mice. TH+ dopaminergic neurons are shown in green, DAPI+ nuclei are shown in blue. (A) Overview of the substantia nigra of 24‐month‐old mice. Scale bar: 200 μm. (B) Higher magnification shows that OPCs are still co‐localized with TH+ neurons in the substantia nigra of 24 months old mice (white arrow). Scale bar: 50 μm. (C) Localization of the substantia nigra. Scale bar: 100 μm. (D) OPCs interact with the somata of TH+ dopaminergic neurons (white arrow) in the substantia nigra of 24‐month‐old mice. Scale bar: 25 μm.

Finally, we chose the very late time point of 24 months, in order to catch changes that might be induced due to aging. In 24 months (24 m) old mice, the dopaminergic neurons of the SNpc are still intermingled with OPCs (Figure 3A,C). Higher magnification images from 24 m old mice show the same close proximity of OPCs with dopaminergic neurons (white arrows, Figure 3B,D) as seen at P4 and P15, indicating that OPC‐dopaminergic neuron interactions in the SNpc persist during development and throughout adulthood. Thus, we did neither find differences in the proportion of TH neurons, nor in OPCs, and also not in their interaction with each other in aged mice.

### 3.2 OPCs and Dopaminergic Neurons as a Percentage of Total Cells in the SNpc

**FIGURE 4:**
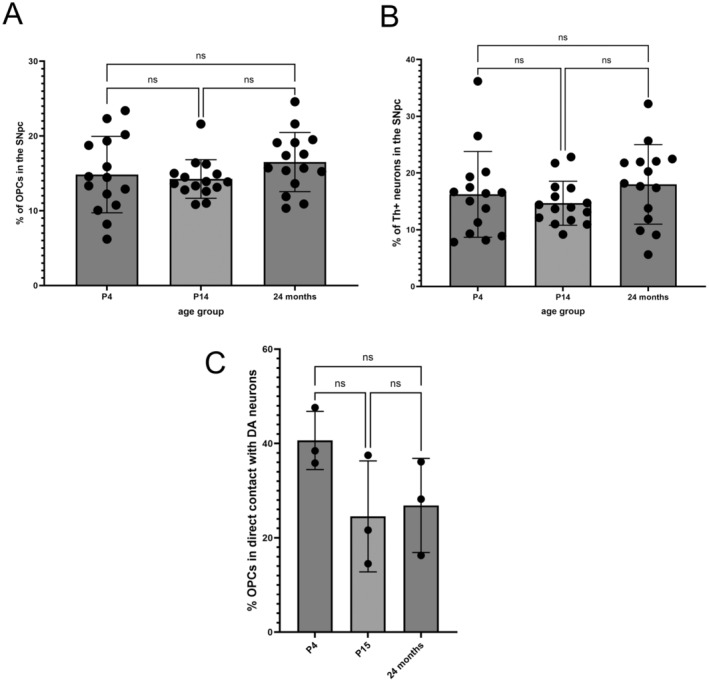
Percentage PDGFRα and TH positive cells in the SNpc. The percentage of (A) OPCs (PDGFRα+) and (B) Dopaminergic neurons (TH+) in the SNpc of wild‐type mice at P4, P15, and 24 months. Positively stained cells were counted as a proportion of total cell nuclei (DAPI+) in the SNpc. 5 images were taken per mouse from *n* = 3 mice at each age group. Comparisons were made using the one‐way ANOVA statistical test. There were no significant differences (ns) between the groups and conditions. The degrees of freedom between groups were 2 and within groups were 42 for both analyses. For OPCs, the ANOVA yielded an *F* value of 1.283 with a *p*‐value of 0.2880, and for dopaminergic neurons, the ANOVA yielded an *F* value of 1.025 with a *p*‐value of 0.3677. Post hoc Tukey's test was applied with corrections for multiple comparisons. The results showed no significant differences between different groups (all *p* > 0.05). (C) The percentage of OPC surface (reconstruction of PDGFRa+ labeling) that is in direct contact with the DA neuron surface (reconstruction of TH+ labeling) at P4, P15, and 24 months of age. *n* = 3 mice each group and 3 images were taken per mouse. The Kruskal–Wallis test was used to compare differences between age groups. Degrees of freedom were 2, H = 3.289, *p* = 0.2321. No corrections for multiple comparisons were necessary.

We defined a strict cut‐off for the SNpc avoiding any overlap between nearby regions and counted all nuclei, OPCs, and dopaminergic neurons. Expressed as a percentage of total cells in the SNpc, we found that OPCs (Figure 4A) account for 14.84% (±5.11 SD), 14.25% (±2.58 SD) and 16.52% (±3.95 SD) of all cells at P4, P14 and 24 m respectively. The dopaminergic neurons account for 16.24% (±7.56 SD), 14.67% (±3.88 SD), and 17.99% (±7.00 SD) of all cells at the same ages (Figure 4B). There is no statistical difference between the percentage of OPCs or dopaminergic neurons between the age groups. The respective numbers for each age group suggest that OPCs and dopaminergic neurons exist in an approximate 1:1 ratio in the SNpc, which remains stable throughout the lifespan. Next, we assessed the extent of contact formation between OPCs and DA neurons in the SNpc. Therefore, we generated a surface from both channels, displaying PDGFRα‐labeling and TH‐labeling, and automatically measured the distance between both surfaces (Figure S2). When comparing the closest interactions (no distance), we found that 40.64% (±6.2) of the entire OPC surface was in direct contact with the surface of Th + neurons. At P15, this amount decreased to 24.55% (±11.8) and stayed stable at 24 months of age, where 26.87% (± 10%) of the entire OPC surface was in direct contact with the TH+ surface representing the DA neurons (Figure 4C).

### 3.3 Myelin‐Distribution in the Midbrain at Post‐Natal Day 15

**FIGURE 5:**
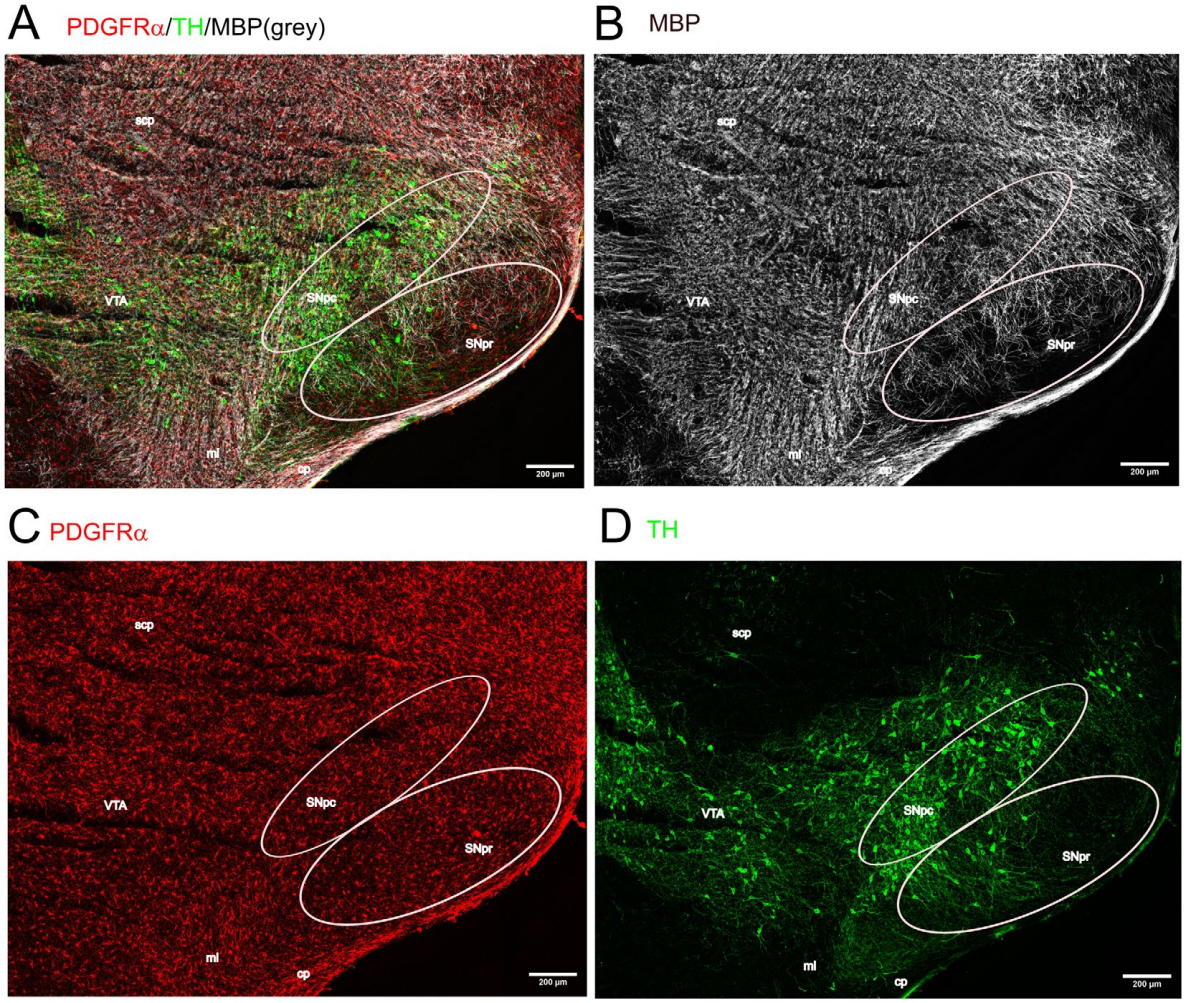
Myelination in the midbrain at P15. The distribution of myelin (MBP, shown in gray) is shown in coronal sections of P15 old mouse midbrain. PDGFRα is shown in red, and TH+ dopaminergic neurons are shown in green. (A) Overlay. Areas containing myelin bundles as well as the SN are labeled. The indicated areas in A are magnified in Figure 6. (B–D) Single channels. Scale bar: 200 μm. cp, cerebellar peduncle; ml, medial lemniscus; scp, superior cerebellar peduncle; VTA, ventral tegmental area.

**FIGURE 6:**
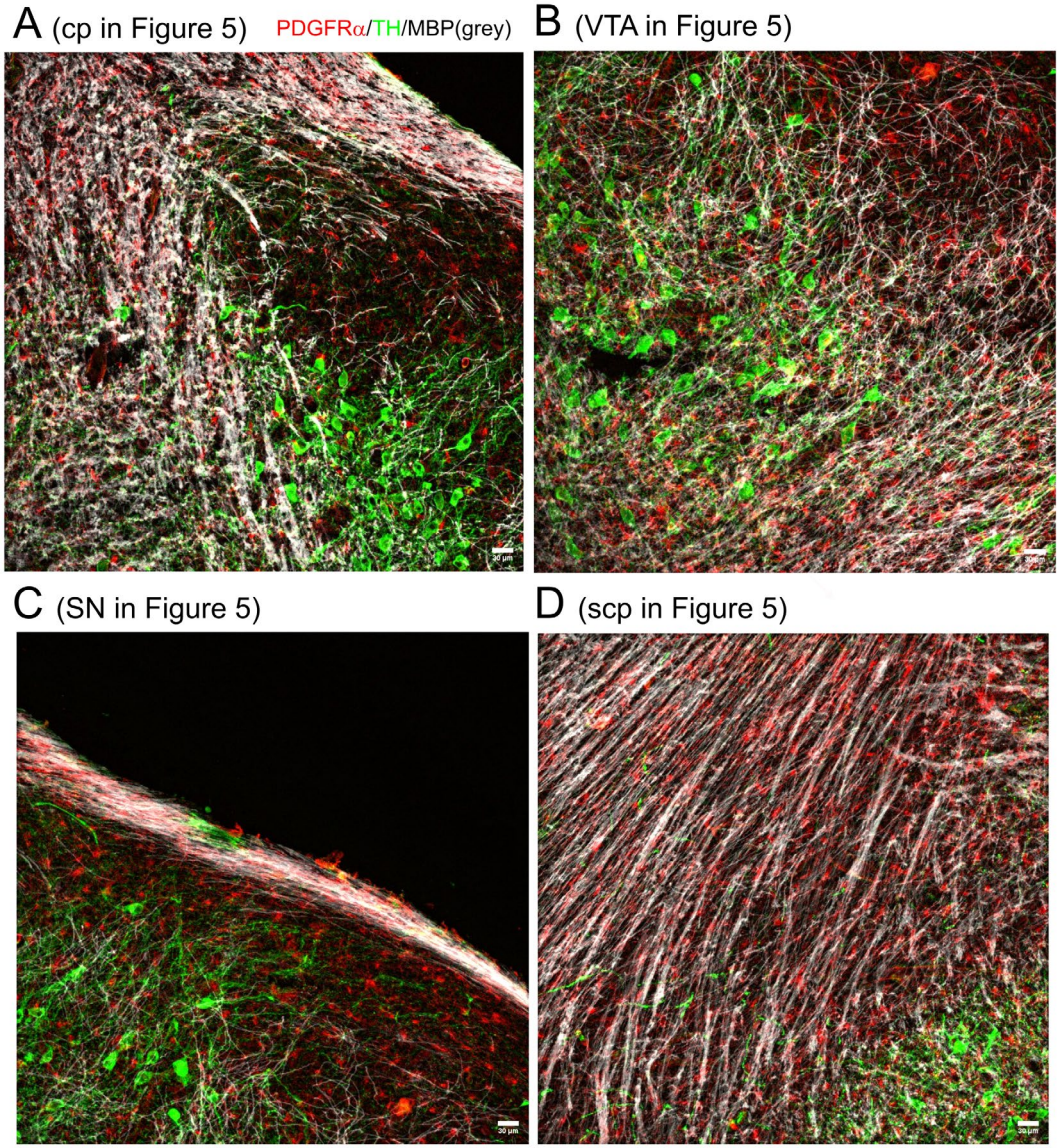
Myelination in the midbrain at P15. The distribution of myelin (MBP, shown in gray) is shown in coronal sections of P15 mouse midbrain. PDGFRα is shown in red, and TH+ dopaminergic neurons are shown in green. (A) Cerebral peduncle (CP) and SN. (B) Thalamic fibers in the ventral tegmental area (VTA) and medial lemniscus (ml). (C) Dopaminergic projections in the substantia nigra pars reticulata (SNpr). (D) Myelinated fibers in the superior cerebellar peduncle (scp). All scale bars indicate 30 μm.

Myelination is generally accepted to be absent at Post‐natal Day 4 (P4) since myelination begins around post‐natal Day 7 (reviewed in (Nishiyama et al. 2021)). At P4, we also did not observe myelination in the midbrain (Figure S1). We therefore focused on the characterization of myelination in the midbrain at P15, the age group where myelin sheaths become clearly visible (Figure 5A). While most of the adjacent areas contain many myelinated fibers, myelination is sparse in the SN (Figure 5A,B). Especially, dopaminergic fibers originating from the SNpc and projecting through the SNpr lack myelination (indicated in Figure 5A, details in Figure 6A,C). Myelin sheaths are numerously present in areas of the midbrain that either contain TH+ dopaminergic neurons such as the VTA and the medial lemniscus (Figure 6B) or do not contain TH+ dopaminergic neurons such as the fibers of the superior cerebellar peduncle (Figure 6D). Regardless of the distribution of myelin, OPCs are distributed evenly throughout the midbrain (Figure 5C).

### 3.4 Differences in the Proportion of OPCs Among All Oligodendroglial Cells in the SNpc Versus SNpr

**FIGURE 7:**
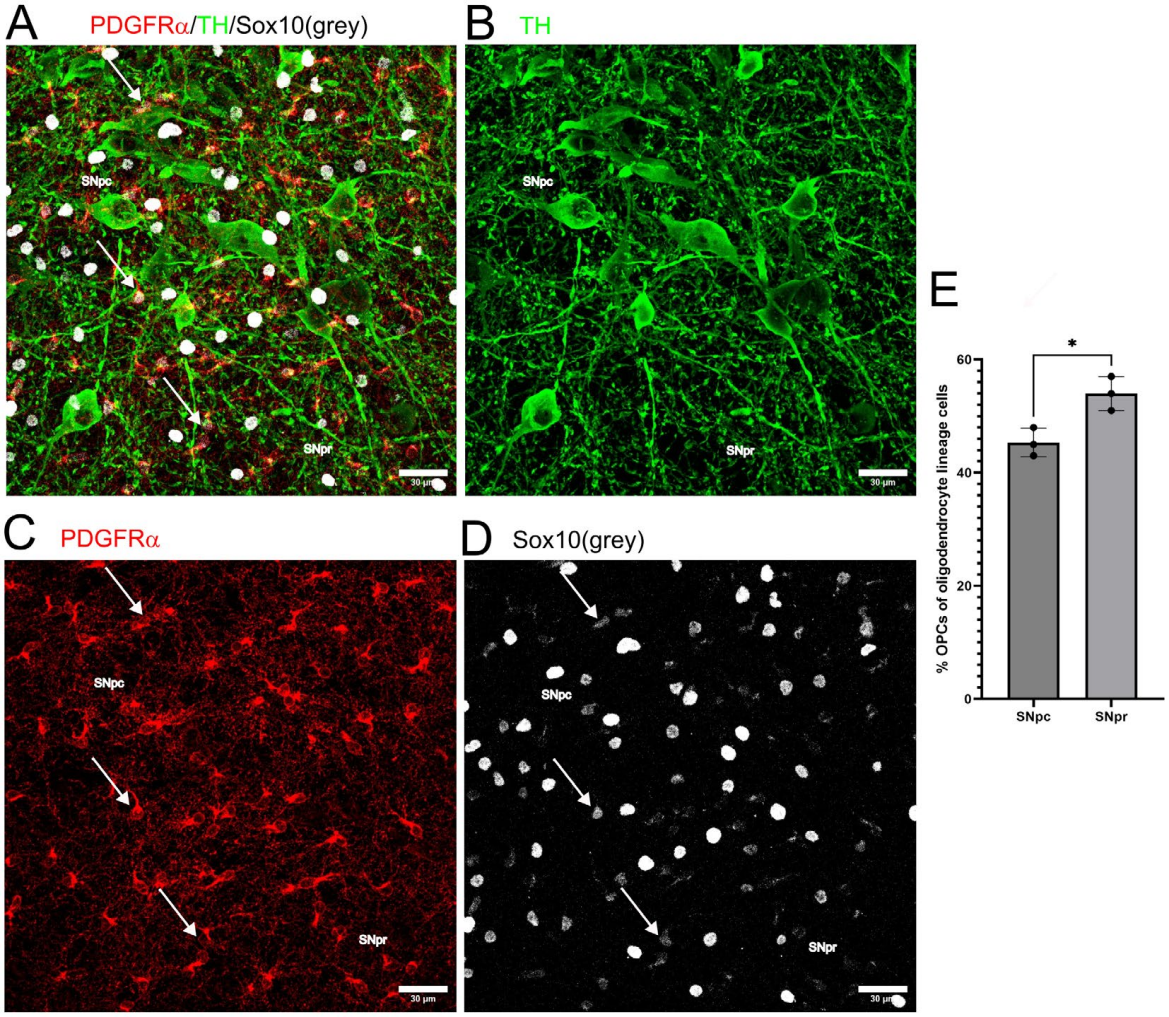
Percentage PDGFRα positive cells among oligodendroglial cells in the SN. The percentage of PDGFRα positive OPCs (shown in red) among all oligodendroglial cells (Sox10, shown in gray) in the SN (identified by TH positive neurons, shown in green) of P15 old mice (A) Overlay. Scale Bar: 30 μm. (B–D) Single channels. Scale Bar: 30 μm. (E) Statistical analysis of the portion of OPCs among all Sox10 positive oligodendrogilal cells in the SNpc (45.33% ± 2.5%) and the SNpr (54% ± 3.0%). Comparison was made using an unpaired two‐tailed *t* test. Degrees of freedom were 4, *t* = 3.833, *p* = 0.0186.

Since there is more myelin in the SNpc as compared to the SNpr, where myelin is mostly absent, we assessed the proportion of OPCs among oligodendroglial cells expressing the transcription factor Sox10 (Figure 7A–D) in P15 old mice. Sox10 appears in the pre‐OPC stage and remains throughout differentiation into oligodendrocytes and in the mature state (reviewed by (Sock and Wegner 2021)). We found that while in the SNpc 45% of all oligodendroglial cells were OPCs, this number was increased to 54% in the SNpr (Figure 7E). These data indicate that about half of the cells from the oligodendroglial lineage further differentiate, while the other half remains in the precursor state. The fact that there is a higher portion of precursors in the SNpr reflects the fact that this specific area is almost unmyelinated. This indicates that these precursor cells exert functions apart from serving as a reservoir for newly forming oligodendrocytes.

To rule out that major changes in the neuronal composition of the SNpc occur during life span that could impact the ratio between OPCs and DA neurons, we analyzed the proportion of TH‐positive DA neurons among all neurons (NeuN‐positive) in this area. There was a slight increase in the percentage of DA neurons among all neurons in the SNpc from 51% at P15 to 60% at 24 months of age (Figure S3) but no major changes in the cellular composition of the SNpc, supporting our observation that the OPC‐DA neuron interaction in the SN is stable during development and aging.

## 4 Discussion

In this study, we describe the distribution of OPCs and dopaminergic neurons and their morphological interactions with each other in the mouse *substantia nigra* across aging for the first time.

The presence of OPCs and oligodendrocytes in the midbrain has been shown before in the midbrain of zitter rats (Sakakibara et al. 2008). Another study showed that NG2‐positive cells were localized close to TH‐positive neurons (Kitamura et al. 2010). Single‐cell RNA sequencing of human brain tissue and human midbrain organoids also showed the presence of OPCs and oligodendrocytes (Wang et al. 2024).

Since SNpc dopaminergic neurons are scarcely myelinated (Braak and Del Tredici 2004; Haddad and Nakamura 2015; Orimo et al. 2011), we were interested in whether OPCs could interact with dopaminergic neurons for purposes that are independent of the capability of OPCs to differentiate into mature myelinating oligodendrocytes. In this study, we show that OPCs are distributed evenly throughout the SNpc and provide evidence of close OPC interaction with the cell bodies of neurons in addition to axons at P4, P15, and 24 months of age. Furthermore, we show that the distribution of OPCs does not coincide with myelin basic protein (MBP) at P15.

We know that there is a close association between OPCs and noradrenergic, tyrosine hydroxylase‐positive neurons in the mouse locus ceruleus (LC) (Seifi et al. 2014). Interestingly, these neurons are also mostly unmyelinated (Aston‐Jones, Segal, and Bloom 1980). In line with these findings, we also observe interactions between OPCs and dopaminergic neurons of the SNpc and can show that the projections from these neurons in the SNpr were unmyelinated.

In our study, we observe a 1:1 ratio of OPCs and dopaminergic neurons in the healthy SNpc, accounting for approximately 40% of all cells in the region. Interestingly, this OPC: dopaminergic neuron ratio in the SNpc remains stable throughout development and aging. Here we show that although the percentage of cells expressing PDGFRα or tyrosine hydroxylase increases slightly from ~15% in the P4 and P15 mice to 20% in the 24‐month‐old mice, the ratio stays the same. These findings are interesting with regard to the well‐described portion of OPCs in the cortex, hippocampus, spinal cord, or corpus callosum: while OPCs comprise 10%–20% of all cells in the juvenile white matter and 5%–9% in the juvenile gray matter of rodents, their number drops to 5% or lower in all of these areas investigated so far in the adult rodent (Dawson et al. 2003; Pfeiffer 2022). We show here that the development of cell numbers in the SN is different, and starts with a relatively high number of OPCs in juvenile mice compared to other gray matter regions (15% of all cells are OPCs at P4 and P15) with even a slight increase at 24‐month‐old mice. Furthermore, we show that OPCs comprise half of all oligodendroglial cells in the SN at P15, indicating that half of the cell lineage was further differentiated. However, from our results, we cannot distinguish whether OPCs are continuously replaced in the SN, or even increase their proliferation during the lifetime of the organism, or whether other cell types are decreased in numbers during aging and OPCs are not. Their high number is nevertheless indicative of an important role of OPCs in dopaminergic neuronal function.

Our finding of a 1:1 ratio of OPCs and dopaminergic neurons in the SNpc, together with the close interaction of OPCs with the dopamine neuron cell bodies in the SNpc, further support the emerging concept that OPCs play an important regulatory and/or supportive role in this region. OPCs have been shown to be important for the maintenance and remodeling of neurons by engulfment and removal of their synapses (Buchanan, da Costa, and Cheadle 2023; Haroon et al. 2024). Seifi et al. (2014) suggest that OPCs may be involved in behavioral adaptions to aversive life events, and may even regulate LC circuits by modulating GABAergic input to noradrenergic neurons thus reducing the release of noradrenaline. The tight connections we observe between OPCs and dopamine neuron cell bodies and axons implicate general biochemical support, which we speculate to involve bioenergetics or regulation of neuronal activity. It is well known that neurons establish synaptic contacts with OPCs and these contacts elicit postsynaptic excitatory potentials in OPCs (reviewed by Thornton and Hughes 2020), which in turn regulate their differentiation into mature oligodendrocytes. In the *corpus callosum*, it was suggested that OPCs react to dopamine released by midbrain‐originating axons, as they express Drd1 and Drd2 transcripts, contributing to myelin plasticity in this area (Caldwell et al. 2023). Conversely, the relevance of OPC interactions in regulating dopaminergic neuron activity is unknown. We describe a close interaction between OPCs and the soma of dopaminergic neurons in the SNpc, not their axon terminals. The input onto dopaminergic neurons in the substantia nigra is mainly GABAergic (Paladini and Tepper 2017). OPCs have been shown to express GABA receptors (Von Blankenfeld et al. 1991) and their activation by GABA release causes activating currents in OPCs (Larson et al. 2016; Lin and Bergles 2004). Further, GABA signaling onto OPCs has been shown to increase OPC proliferation and suppress the generation of oligodendrocytes in cerebellar white matter (Zonouzi et al. 2015). Whether there is a direct link between the release of GABA and the increased number of OPCs in the SN as compared to other gray matter areas remains to be determined. It is currently unclear which function these OPC contact formation with the neuronal soma may have. It is possible that this close interaction with the soma has a regulatory, or even neuroprotective function, but whether it is exerted by physical interference with projecting neurons, synaptic reorganization or the exchange of signaling molecules needs further investigation.

The dopaminergic neurons of the SNpc have a particularly high energetic demand due to their pacemaking activity, active calcium pumping, and poorly conductive, large axonal arbors. This contributes to high levels of oxidative stress via mitochondria and dopamine oxidation (Burbulla et al. 2017; Gonzalez‐Rodriguez et al. 2021). Interestingly, in the 6‐hydroxydopamine (6‐OHDA)‐injected PD mouse model, OPCs were located close to surviving dopaminergic neurons (Kitamura et al. 2010), indicating a protective effect of NG2 glial cells in this model. Thereby, OPC interactions with dopamine neurons in the degenerating striatum and SNpc (Kitamura et al. 2010) follow the processes first and cell body last model of PD neurodegeneration described by Gonzalez‐Rodriguez et al. (2021). Future investigations addressing the physiological role and consequences of this close interaction between DA neurons and OPCs we described here are necessary to define its role and importance in the midbrain. In this context, the relevance of OPC density in health and disease is the subject of ongoing research.

## Author Contributions

**Julia C. Fitzgerald:** conceptualization, data curation, formal analysis, funding acquisition, resources, supervision, writing – original draft, writing – review and editing. **Ying Sun:** conceptualization, data curation, formal analysis, methodology, writing – review and editing. **Frederek Reinecke:** conceptualization, data curation, formal analysis, methodology. **Elisabeth Bauer:** conceptualization, data curation, formal analysis. **Olga Garaschuk:** conceptualization, funding acquisition, resources, writing – review and editing. **Philipp J. Kahle:** conceptualization, funding acquisition, resources. **Friederike Pfeiffer:** conceptualization, data curation, formal analysis, funding acquisition, project administration, resources, supervision, writing – original draft, writing – review and editing.

## Ethics Statement

All experiments including mice were performed in accordance with the German Animal Welfare Law and the allowance of the (local) authorities (Regierungspräsidium Tübingen) under the approval reference number PY01/21G and N14/20G. No human samples are included in this study.

## Conflicts of Interest

The authors declare no conflicts of interest.

### Peer Review

The peer review history for this article is available at https://www.webofscience.com/api/gateway/wos/peer‐review/10.1111/jnc.16298.

## Supporting information


Figure S1‐S3.

**Movie S1:** P4 Voxel size X: 0.270 Y: 0.270 Z: 0.345; Size 413 × 427 × 73.

**Movie S2:** P15 Voxel size X: 0.271 Y: 0.271 Z: 0.346; Size 1024 × 1024 × 5.

## Data Availability

The datasets generated during and/or analyzed during the current study are available in the Mendeley repository, Fitzgerald, Julia C. (2024), “OPC_DA neuron_MBP_Midbrain,” Mendeley Data, V1, doi: 10.17632/672pp73xnd.1 together with a detailed immunofluorescence protocol for reproduction.
