# Supplementary material for: Interactions of Oligodendrocyte Precursor Cells and Dopaminergic Neurons in the Mouse Substantia Nigra
Source: J Neurochem. 2025 Jan 27;169(1):e16298. doi: 10.1111/jnc.16298 (PMC11773302; doi:10.1111/jnc.16298)
Supplement: Supplementary file 1 — Figure S1‐S3. [file JNC-169-0-s003.pdf]

Interactions of Oligodendrocyte Precursor Cells and Dopaminergic Neurons in the Mouse Substantia Nigra

Julia C. Fitzgerald[1], Ying Sun[2], Frederek Reinecke[2], Elisabeth Bauer[1], Olga Garaschuk[2], Philipp J. Kahle[1,3,4], Friederike Pfeiffer[2*]

Supplementary Figures

Supplementary Figure 1

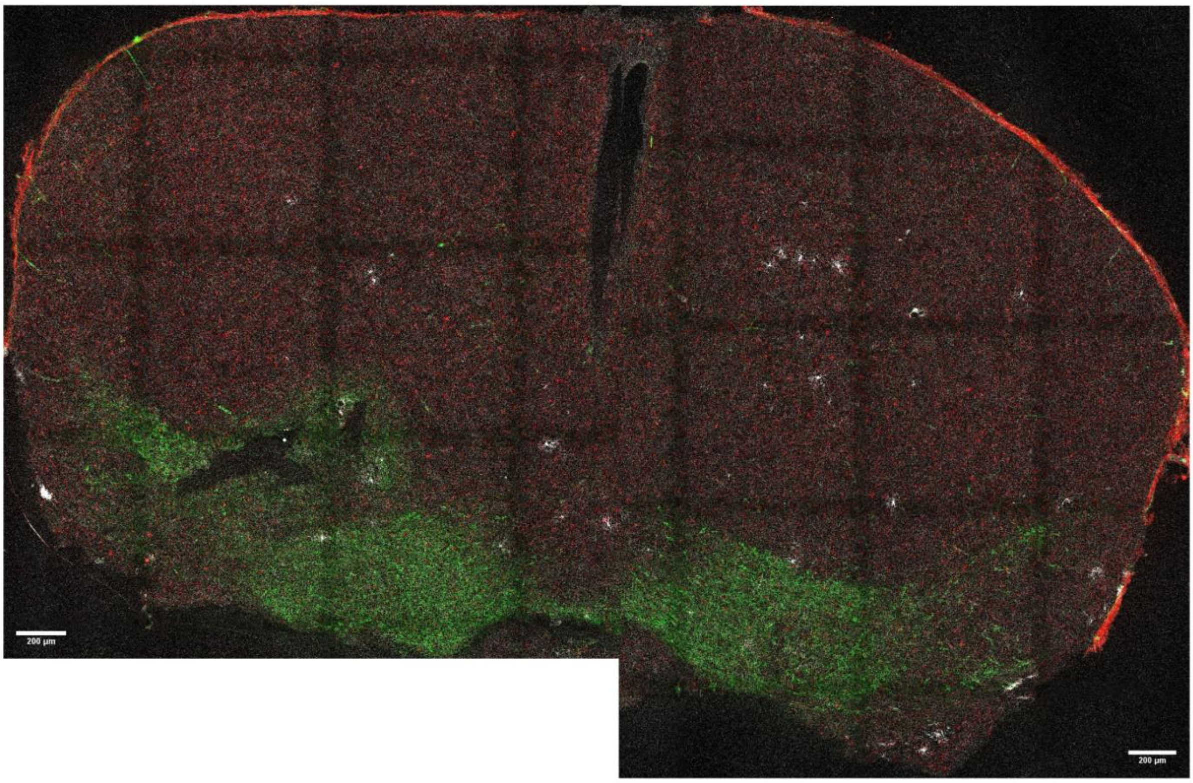

Supplementary Figure 1: Coronal section through the midbrain at P4.

PDGFRα is shown in red, TH+ dopaminergic neurons are shown in green, MBP is shown in grey. At P4, some maturing oligodendrocytes appear that express MBP, but no myelin sheaths are detectable yet. Scale bars represent 200µm.

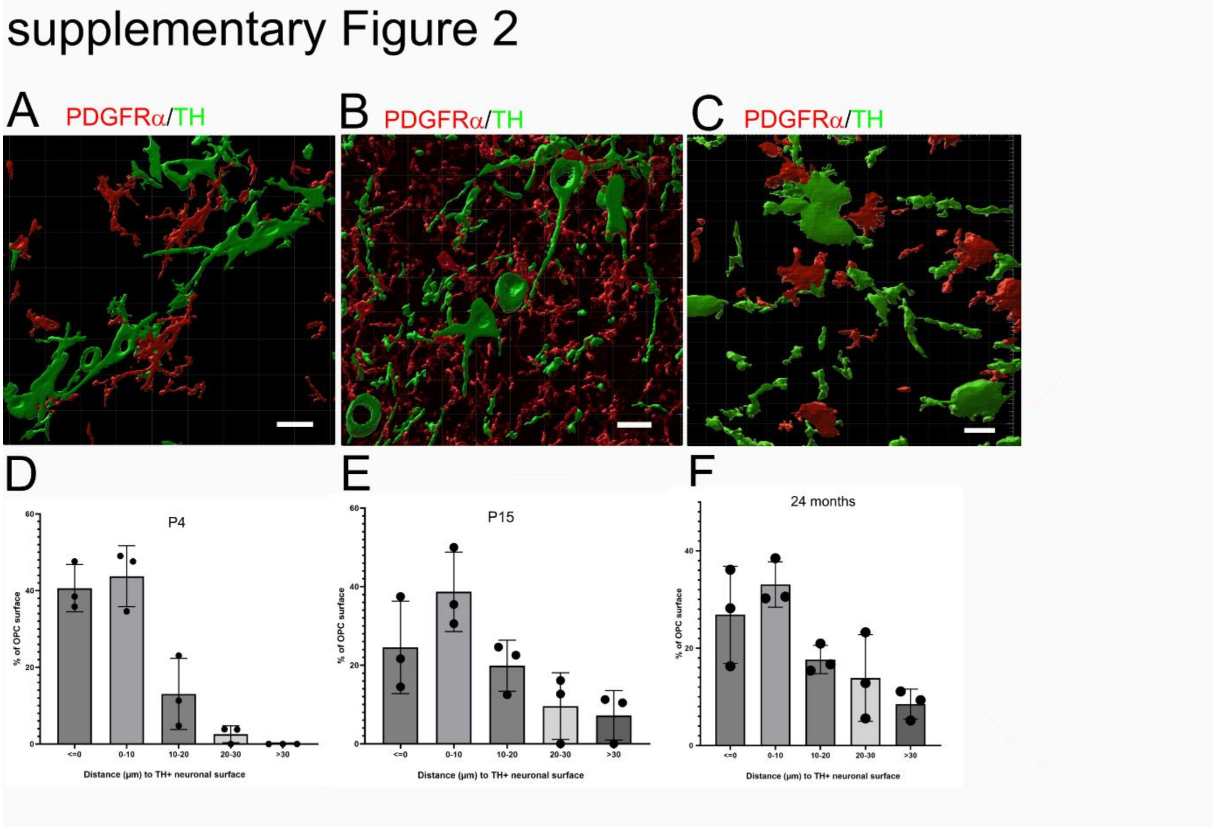

Supplementary Figure 2: Quantification of contacts between OPCs and DA neurons in the SN.

Surface reconstructions of PDGFRa positive OPCs (in red) and TH positive neurons (in green) and assessment of the distance between them. A: SN of P4. Scale Bar: 20µm. B: SN of P15. Scale Bar: 20µm. C: SN of 24 months. Scale Bar: 15 µm. D: Graph showing binned distances between both surfaces at P4. E: Graph showing binned distances between both surfaces at P15. F: Graph showing binned distances between both surfaces at 24 months. The statistical comparison of direct contact (<=0) between the age groups is shown in Figure 4C.

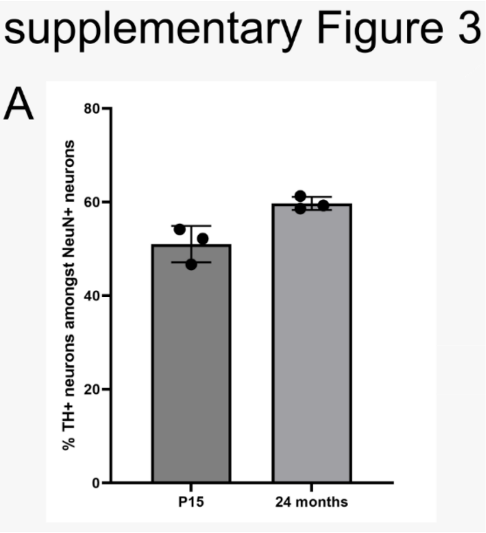

Supplementary Figure 3: Percentage of TH+ neurons amongst all neurons (NeuN) in the SNpc

Analysis of the proportion of dopaminergic neurons amongst all neurons in the SNpc. TH+ neurons comprise 51% of all (NeuN+) neurons at P15 and 60% of all (NeuN+) neurons at 24 months. Data was acquired from one mouse per group to show that there is no specific loss of DA neurons in the SNpc. No statistical comparison was performed.
